# Antibiotic use and resistance patterns at Rumphi District Hospital in Malawi: a cross-sectional study

**DOI:** 10.1186/s12879-024-09333-w

**Published:** 2024-04-26

**Authors:** Brany Mithi, Mosen Luhanga, Felix Kaminyoghe, Francis Chiumia, Daniel L. Banda, Lottie Nyama

**Affiliations:** 1 School of Global and Public Health, Kamuzu University of Health Sciences (KUHeS), Blantyre, Malawi; 2 Department of Medical Laboratory Sciences, Kamuzu University of Health Sciences (KUHeS), Blantyre, Malawi; 3 Department of Pharmacy, Kamuzu University of Health Sciences (KUHeS), Blantyre, Malawi; 4 Ministry of Health, Rumphi District Hospital, Rumphi, Malawi; 5 Pharmaceutical Society of Malawi (PHASOM), Lilongwe, Malawi

**Keywords:** Antibiotic resistance, Point Prevalence Survey, Multi-drug resistance, Malawi

## Abstract

**Background:**

Overuse of antibiotics is a key driver of antimicrobial resistance (AMR) world-wide. Malawi continues to report rising cases of AMR among both in-patients and out-patients. We investigated antibiotic use and resistance patterns among patients with suspected first line antibiotic treatment failure at Rumphi District Hospital, Malawi.

**Methods:**

We used a cross-sectional study design in which records of patients data on culture and antimicrobial sensitivity tests were extracted, alongside treatment history from 2019 to March, 2023, retrospectively. We also included findings for point prevalence survey (PPS) conducted within four hospital wards in June, 2022 by a well-trained multi-disciplinary team from within the hospital. The data was analyzed for antibiotic use, characterization of pathogens and their susceptibility patterns using Microsoft excel and STATA-14 software.

**Results:**

A total of 85 patients’ data records were reviewed on antibiotics resistance pattern in which 54 (63.5%) were females. Patient antibiotic history captured indicated Metronidazole (23%), Gentamycin (20%) and Doxycycline (23%) as the most frequently used antibiotics among clients referred for microbiological investigations. Among locally available antibiotics with over 50% sensitivity were Chloramphenicol (61%), ciprofloxacin (55%), and ceftriaxone (54%). Penicillins were among antibiotics with highest resistance: ampicillin (100%), amoxyclav (90%), Piperacilin-tazobactam (63%). The majority of patients came from STI clinic and presented with genital discharges 44% (*n* = 39). Over 80% of the isolated *N. gonorrhoeae* exhibited a reduced susceptibility to gentamycin. Prevalence of Methicillin resistant staphylococcus Aureus (MRSA) was 46% and were mostly isolated from wound pus. Among 80 data records of the patients reviewed during PPS, Ceftriaxone (54.3%) and Metronidazole (23.3%) emerged as the most frequently used antibiotics in the wards which were prescribed empirically without a microbiological indication.

**Conclusion:**

In this study setting, we observed high use of watch antibiotics along with problem of multi-drug resistant infections in patients experiencing clinical failure in a variety of clinical syndromes. The findings underline the need to revamp diagnostic microbiology to increase the uptake of antimicrobial susceptibility testing to guide specific prescriptions of broad-spectrum antibiotics in the watch list.

## Introduction

Antimicrobial resistance (AMR) is a silent pandemic and one of the top 10 public health challenges worldwide as described by World Health Organization (WHO) [1]. The rising incidence of multi-drug resistant (MDR) microbes as a result of irrational and overuse of antibiotics contribute significantly to morbidity and mortality among hospitalized patients [2]. AMR threatens to erode the ability to treat infections with the available antibiotics as the pace of developing new antibiotics by the pharmaceutical industry has been quite slow the past two decades [3]. The immediate implications of antibiotic resistance include death and disability, extended hospital stays due to prolonged sickness, need for expensive therapies, and rising healthcare expenditure [2, 4, 5]. Consequences of AMR are quite grave in low and middle income countries (LMIC) as they cannot afford new expensive antibiotics [6, 7].

Malawi continues to report rising cases of AMR among both in-patients and out-patients. Several studies have been conducted in the past two decades, signifying the emerging superbugs [7, 8]. A retrospective study conducted at Queen Elizabeth central hospital in Malawi from 1998 to 2016, reported rapid expansion of extended spectrum β-lactamases (ESBL) and fluoro-quinolone resistance among gram-negative pathogens, and the emergence of methicillin resistant staphylococcus aureus (MRSA) [9]. A prospective study conducted at Kamuzu central hospital in Malawi reported resistance to several first line antibiotics for treating bacteremia and other life threatening infections [10]. A similar prospective study at queen elizabeth central hospital (QECH) that used whole-genome sequencing and cox regression models to estimate effect of resistance to third generation cephalosporins reported increased mortality and longer hospital stays in patients with bloodstream infections in Malawi [11]. The resistance patterns observed in central hospitals may not be the same compared with secondary referral facilities as the treatments and as well as the infection control and prevention practices greatly vary. However, of the commonly used antibiotic in both central and secondary hospitals in Malawi are third generation cephalosporins whose resistance rates are reportedly high [12]. Resistance to cephalosporins present a big challenge as they are used as first line and last line antibiotics in many secondary health facilities in Malawi.

A cross sectional study conducted among households in densely populated townships in Malawi revealed high usage of antibiotics such as Amoxicillin, Erythromycin and Cotrimoxazole [12]. These are among antibiotics with diminished effectiveness reported in many studies on antimicrobial resistance pattern in Malawi and sub-Saharan region [13, 14]. As reported by the Global Research on AntiMicrobial resistance (GRAM), In 2019 alone, Malawi registered about 3600 deaths attributable to AMR [15]. This signifies the burden of bloodstream infections that pose a challenge to treat especially in resource-limited setting. At Rumphi district hospital, AMR has led to an upsurge of clinical failures with a third of clients with sexually transmitted infections (STI) returning to the facility after failing to respond to first line antibiotic treatment.

As a way of supplementing global efforts to combat AMR, observing AMR patterns allows for timely identification of emerging superbugs as well as developing new interventions, including updating the available national standard treatment guidelines [16]. As part of ensuring full participation in the fight against AMR, Rumphi district hospital antimicrobial stewardships (AMS) committee has been conducting series of AMS activities including participating in global point prevalent survey (GPPS); reviewing and presenting laboratory findings on AMR and as well as facilitating awareness campaigns to educate healthcare workers on appropriate antibiotics prescribing practices. Such activities are well supported by an AMR coordinating center (AMRCC) stationed at the public health Institute of Malawi (PHIM) [17–19].

Monitoring of antibiotic use and resistance patterns underpin the effective implementation of AMS interventions in combatting AMR [20]. To date, few studies have assessed antibiotic use and antimicrobial resistance pattern in secondary referral hospitals in Malawi. Most studies on AMR have been focused on in-patients in tertiary hospitals. In comparison with tertiary hospitals, secondary hospitals have inadequate staff and fewer resources to support precise diagnosis and rational use of antibiotics. However, there is limited data to provide insights on the antibiotic medicine related problems in secondary hospitals in Malawi. This retrospective study investigated frequency of antibiotics use and resistance pattern at Rumphi district hospital, a secondary referral facility In Malawi. Findings for this study will help the hospital AMS team to better understand the burden and relationship between AMR and antibiotics overuse. The goal is for hospital AMS teams to co-design effective interventions including formulation of antibiogram which provide a reference for empirical antibiotic prescriptions.

## Methods

### Study design

We conducted a cross-sectional study of antimicrobial prescribing patterns among hospitalized patients and analyzed laboratory antimicrobial susceptibility tests results for patients who presented with suspected antibiotic treatment failures. We further made a review of PPS findings to ascertain prevalence of antibiotic use and prescribing practices in hospital wards. PPS are snapshot audits across a whole hospital or selected wards, made at regular intervals to track trends and show the number of people taking antimicrobials at a given point in time.

### Study site

The study was conducted at Rumphi District Hospital in the northern part of Malawi. The district covers an area of 4,769 km², with a population of 128 360 people. The facility was chosen because it has an active AMS committee capable of conducting point prevalence survey. Moreover, it has an accredited laboratory with the capacity to conduct culture and AST. The study site is a secondary referral facility for 14 health centers and 3 community hospitals. Patients in peripheral sites requiring culture and AST tests are referred to Rumphi District Hospital laboratory.

### Study population

Secondary AST data records for both in-patients and out-patients whose majority are cases that never responded to first line antibiotic treatment during the period of 2019 to 2023, were analyzed in this study. Determination of treatment failure was based on the clinician’s judgment and was recorded in the patient case notes as persistence or worsening of signs and symptoms during the course of antibiotic treatment. Data with missing values for age, sample type or results for culture tests were excluded. The second set of records were files for hospitalized patients reviewed during PPS as described in the subsequent paragraphs.

### Bacterial identification and drug susceptibility testing

Antibiotic susceptibility testing (AST) on clinical isolates over the period of 2019 to 2023, were done using Kirby-Bauer Disk Diffusion Susceptibility Test protocol while Antibiotics selection for AST and interpretation was done according to the European Committee on Antimicrobial Susceptibility Testing (EUCAST) guide. Patient clinical and antibiotic history recorded on their lab request forms or health passports was documented in the excel sheet together with other relevant information. Different culture media used depending on the nature of the sample and suspected pathogen included Blood agar, Chocolate agar, Macconkey agar, XLD agar, CLED, TCBS and Muller Hinton agar. Gram stain was done on all clinical isolates from culture media incubated for atleast 18 hrs. During identification stage, several biochemical tests were used to identify clinical isolates. Bacterial identification was followed by AST which was done using chocolate agar, blood agar or Muller Hinton depending on the type of bacteria identified.

### Data collection procedure and analysis

#### Point prevalent survey data

Hospital/ward/patient-level data was collected in June 2022 using standardized GPPS patient and ward forms (see in the appendix). The ward form extracted denominator data which comprised all inpatients present in the specific ward before 8 A.M. The patient form captured the numerator data of all admitted inpatients receiving an “active/ongoing” antimicrobial prescription at 8 A.M on the day of the GPPS. Data extracted include details on antimicrobial agents, indications, diagnosis and a set of quality indicators such as stop/review date and compliance to the guidelines. Data was de-identified during extraction and uploaded in specific portal for analysis at Antwerp University (AU) in Belgium. The hospital antimicrobial stewardship team received analyzed results from AU after two months through email. The results received also included the average outcome for five facilities in Malawi.

#### AST data extraction and analysis

Data records for AST were collected from microbiology electronic register retrospectively, covering the period of 2019 to May, 2023. The extracted data was de-identified in excel sheet and it contained information such as date results were reported, sex, department, sample type, antibiotic history, type of pathogen, antibiotic susceptibility pattern results for each antibiotic tested with their respective outcome.

#### Determination of antibiotics sensitivity proportions

Each antibiotic sensitivity test result on a particular pathogen had one of the following outcome; Resistant (R), Intermediate (I), or Sensitive (S). The percentage proportion for each of the three outcomes of the resistance pattern against the bacterial isolate was determined by STATA 14 software program. Overall, an antibiotic with higher proportion of ‘R’ compared to ‘S’ was considered ineffective. Where an antibiotic had more ‘S’ compared to ‘I’ or ‘R’, it was considered effective. Results were presented inform of bar chats with each antibiotic indicating two consecutive sensitivity patterns.

#### Reliability of the results

Rumphi district hospital laboratory is accredited by Southern Africa Development Community Accreditation Service (SADCAS). The laboratory has been implementing quality management systems (QMS) for the past twelve years. It has well qualified and well trained laboratory scientists capable of conducting culture and AST. Besides, the laboratory is involved in a number of proficiency testing schemes for bacteriology such as EQuAFRICA and the National Health Laboratory Service (NHLS). Bacteria identification and interpretation of AST results is done by atleast two laboratory scientists working in the microbiology section.

To ensure high quality and reliable findings for PPS, a multi-disciplinary team comprising a nurse, doctor, pharmacist, lab scientist and infection, prevention and control (IPC) coordinator underwent a three days training orientation, followed by a pretest phase for data collecting tools. The aim was to identify challenges and find a solution before commencement of the study. The online data forms for filling data had several quality checks including dosage, units, and that any data with inconsistence variables or errors was instantly rejected. Data analysis was done by experts at the University of Antwerp hence quality is guaranteed.

## Results

### General antibiotic resistant patterns

A total of 85 patients’ data records from microbiology register were reviewed. The average age was 26 years, with the majority of the clients being female 54 (63.5%). The majority of patients came from STI clinic and presented with genital discharges (*n* = 39). Other common clinical syndromes reported include gastrointestinal symptoms 27% (*n* = 24), abscess and wound pus 15% (*n* = 13), dysuria and hematuria 8% (*n* = 7) and persistent fevers 6% (*n* = 5) (Table 1).

### Antibiotic treatment frequency (%)

We analyzed the antibiotic history records of patients documented on the laboratory requisition forms and health passports for culture and AST. Among the most commonly used antibiotics were Metronidazole 41 (27%), gentamycin 34 (22%), Doxycycline 28 (19%) and cotrimoxazole 12 (10%). STI and cervical cancer screening clinics contributed more clients for culture and AST. (Table 1)

### Characterization of clinical isolates

Several clinical pathogens were isolated from patients presenting with different clinical syndromes. The common bacterial isolates included *Neisseria gonorrhoeae (n = 21), Staphylococcus sp* (*n* = 12), and *Samonella typh* (*n* = 6) **(**Table 1**).** Several isolates of *N. gonorrhoeae* from genital discharges were resistant to gentamycin but sensitive to ceftriaxone. Staphylococcus resistances to cefoxitin, an indicator for MRSA, were also recorded. Both MRSA and highly resistant pseudomonas aeruginosa (*n* = 2) were common pathogens isolated from infected wound pus of hospitalized patients.

**Table 1:** Laboratory patients’ records (A) clinical syndromes and their respective bacterial isolates, (B) Summary of antibiotic history

A. Clinical syndromes and respective common bacterial isolates			
Clinical syndromes	(*n*)/ (%)	Bacterial isolates	(*n*)/ (%)
Genital discharges	39 (44%)	Neisseria gonorrhoeae	21 (40.5%)
Wound/obsess	13 (15%)	Staphylococcus sp	12 (75.0%)
		Pseudomonas aeruginosa	2 (12.5%)
GI symptoms	24 (27%)	Samonella typh	6 (32.0%)
Dysuria/hematuria	7 (8%)	Klebsiella pneumoniae	3 (42.9%)
		Escherichia. coli (E.coli)	2 (28.5%)
		Staphylococcus saprophyticus	1 (14.3)
Persistent fever due to suspected blood infection	5 (6%)	Hemophilus influenzae b	1 (100%)

B. **Overall patients antibiotic history records**			
**Common Antibiotics**	**(n)/ (%)**		
Metronidazole	41 (30%)		
Gentamycin	34 (24%)		
Doxycycline	28 (20%)		
Ceftriaxone	16 (12%)		
Ciprofloxacin	9 (6%)		
Benzyl-penicillin	7 (5%)		

**Fig. 1:**
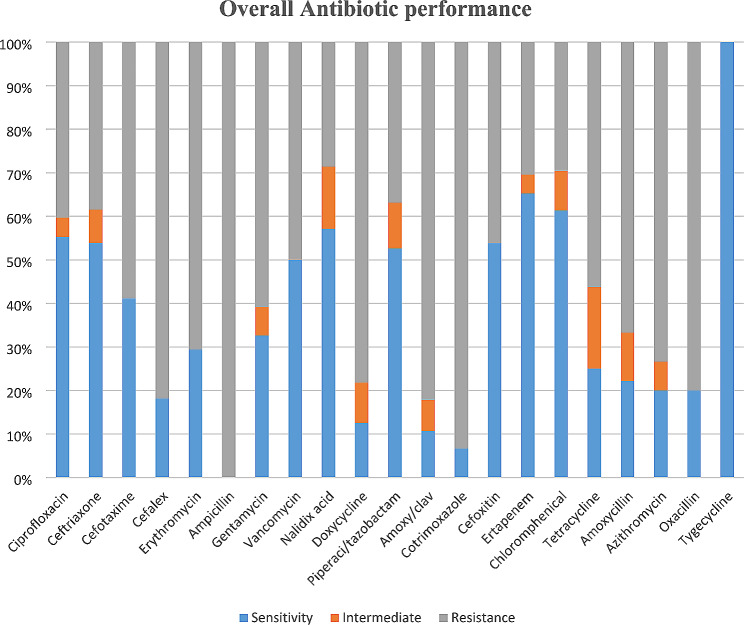
overall antibiotics performance on clinical isolates from non-responsive patients to first line treatment

There were 19 different antibiotics from nine major classes; tetracyclines, penicillins, aminoglycosides, macrolides, cephalosporins, floroquinolones, carbapenens, chloramphenicol and Sulfonamides. Tigecycline emerged the most effective antibiotic with 100% sensitivity while ampicillin registered 100% resistance. Six out of 19 (32%) antibiotics had greater than 50% sensitivity (ciprofloxacin 55%, ceftriaxone 54%, piperacilin/tazobactam 53%, ertapenem 65%, chloramphenicol 61% and Tigecycline 100%). chloramphenicol, ertapenem and Tigecycline had the highest sensitivity in the ascending order. Performance of common antibiotics for treating STIs was quite poor with gentamycin and doxycycline registering sensitivity of 39% and 12% respectively (Fig. 1).

### Point prevalence survey (PPS) results

**Fig. 2:**
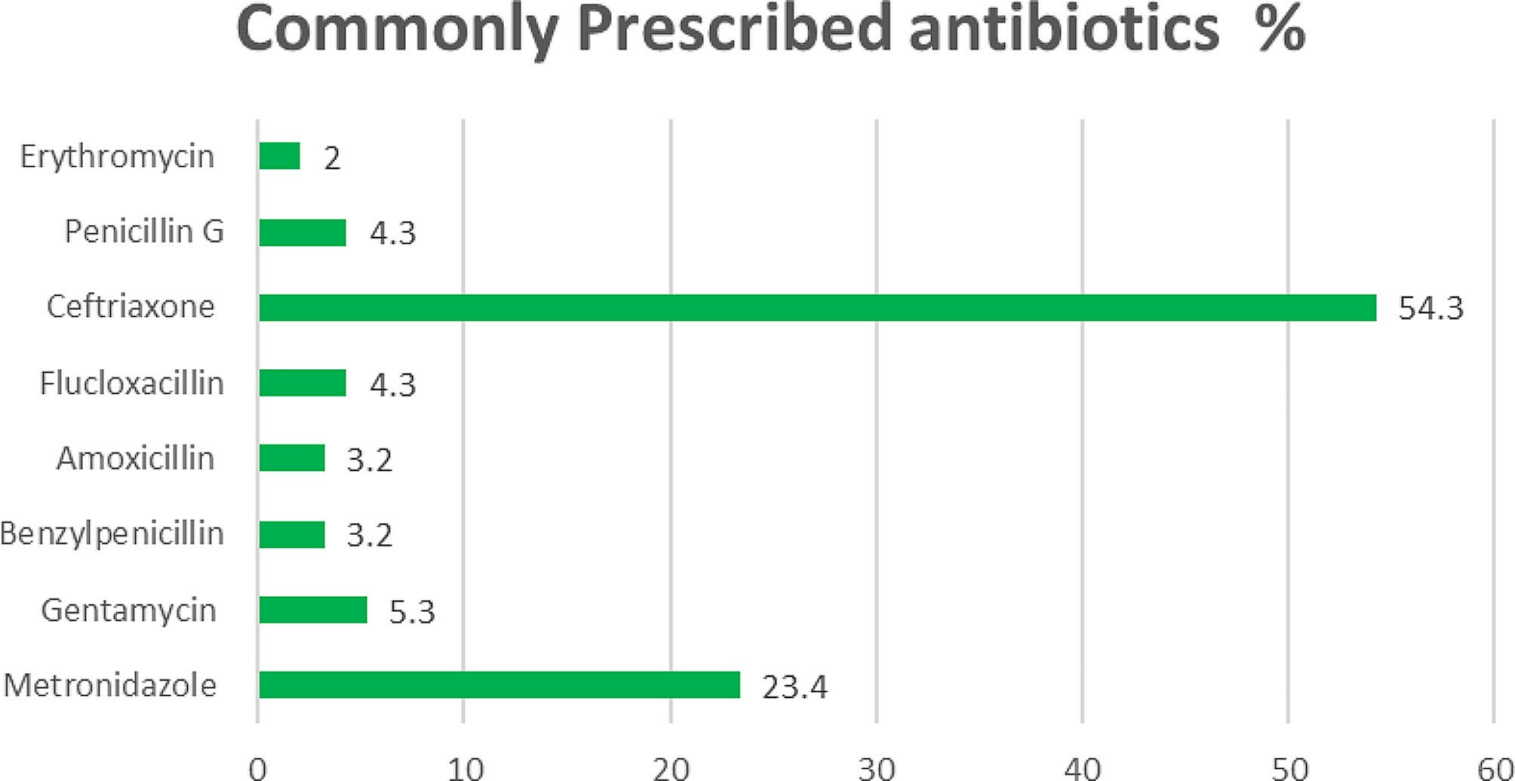
PPS findings indicating commonly prescribed antibiotics in the ward

Regarding PPS, our interest was to determine frequency of antibiotics use as well as compliance with standard guidelines. The findings revealed that ceftriaxone (54.3%) and metronidazole (23.3%) were the most commonly used antibiotics in the wards^1^. Other antibiotics were gentamycin (5.3%), flucloxacillin (4.3%) and penicillin G (4.2%) (Fig. 2). Stockouts of essential antibiotics such as ceftriaxone and penicillin G were reported during PPS data collection.

We further evaluated performance indicators for prescribing practices in the wards which we compared with overall national performance. There were 4 tertiary hospitals and one secondary referral hospital included in the national performance. Stop/review dates for medical, surgical and ICU cases were all above 90%. However, compliance to national treatment guidelines and indicating review notes were poor when compared at national level. Only 39% of patient files had review notes indicated as compared to 93% at a country level for medical patients. Guideline compliant in medical ward was at 73.3% (*n* = 22) against 88.6% at national level. We failed to make a meaningful comparison for ICU as the sample size was quite small (*n* = 2) (Table 2).

**Table 2:** Indicator measures for prescribing antibiotics in the hospital wards

	Our hospital 2022 –P2		Country	
**Medical**	**N**	**%**	**N**	**%**
Reasons in notes	16	39.0	797	93.4
Guidelines missing	0	0.0	8	0.9
Guideline compliant	22	73.3	526	88.6
Stop/review date documented	38	92.7	631	74.0
**Surgical**	**N**	**%**	**N**	**%**
Reasons in notes	15	36.0	224	80.6
Guidelines missing	0	0.0	12	4.3
Guideline compliant	23	74.2	153	81.4
Stop/review date documented	48	98.0	235	84.5
**ICU**	**N**	**%**	**N**	**%**
Reasons in notes	2	50.0	45	80.4
Guidelines missing	0	0.0	1	8.1
Guideline compliant	2	66.7	29	85.3
Stop/review date documented	4	100.0	38	67.9

## Discussion

In this study we characterized common bacterial isolates and determined the antibiotic resistance pattern as well as the frequency of antibiotics use at a secondary referral hospital in Malawi. A cross-sectional data records review was conducted: 80 records for in-patients in the hospital wards and 85 separate records of AST patients’ results, along-side antibiotic history from the microbiology register in the laboratory. Study findings demonstrate that AMR remains a challenge that demands effective AMS multi-faceted interventions such as one health approach.

Both the PPS and the patients’ antibiotic history records from the laboratory registers revealed frequent use of antibiotics classes including cephalosporins, tetracyclines and penicillins. Such findings are in consistent with other studies conducted in South Africa and Uganda [21, 22]. Overall, the resistant pattern of such antibiotics has shown to be considerably high in Malawi based on previous studies and current national AMR reports [10, 11, 15]. Shortage of antibiotics as well as unrevised guidelines partly contribute to irrational use of antibiotics in health care facilities in Malawi and sub-Saharan region [10, 20, 22]. Often times, empirical antibiotic prescriptions are made without any microbiological indication as most healthcare facilities do have limited diagnostic capacity. Indiscriminate use of broad spectrum antibiotics leads to extremely high healthcare costs due to an increase in hospital admissions and drug usage [4].

The PPS also reported prescribing indicators such as poor adherence to the available standard treatment guidelines (< 75% vs. > 80%) as well as poor documentation of review notes (< 40% vs. > 80%) when compared with the overall national PPS results. This is evidence of existence of stewardship implementation challenges in district health facilities unlike in tertiary facilities which are the centers of excellence in AMS. Compared with PPS from Uganda there was no significant difference interms of usage of watch-classified antibiotics (65% vs. 74%), where ceftriaxone topped the list [20]). Integration of AMS programmes in the current practices is therefore crucial. Local experts and frontline stewards who are well knowledgeable about health systems and the existing contextual barriers can play a crucial role in developing and tailoring AMS tools based on the specific health facility needs [23].

The consistently decline in sensitivity of the most locally used antibiotics including ciprofloxacin, ceftriaxone, gentamycin, doxycycline, and ampicillin signifies the growing resistances of bacterial pathogens due to overuse over the past 3 decades [24, 25]. According to the findings of the PPS, ceftriaxone and metronidazole emerged as the leading antibiotics prescribed in the wards. As observed in the antibiotic history of patients with genital discharges, three sets of antibiotics (metronidazole, gentamycin and doxycycline) were frequently prescribed as they form the main treatment package in the syndromic management approach to STI [15, 16]. The findings are in agreement with other studies conducted in sub-Saharan African region which established penicillins, ceftriaxone and metronidazole to be the most prescribed antibiotics with an extended prescribing of up to 6 days for surgical patients [26].

High use of ceftriaxone, metronidazole and other beta-lactam antibiotics in the wards is of great concern. The implications are quite grave. The emergent of highly resistant burgs will likely render our last line treatments ineffective [10, 21, 22]. Hence, the urgent need to tackle inappropriate antibiotic use from all fronts. Probably the one health approach is such an ideal strategy as AMR affect humans, animals and the environment [27].

In our study, we further observed less commonly used vancomycin and chloramphenicol antibiotic performing much better against some resistant bacterial strains. This is in an agreement with other studies that found chloramphenicol maintaining its efficacy against eye infections compared to tetracyclines and floroquinolones [28]. Although most facility no longer use such drugs over the growing concern of toxicity, randomized controlled trials (RCTs) have demonstrated that such antibiotics are as safe as treatment alternative for short antibiotic courses [29]. Government policy supporting rotation of certain broad-spectrum antibiotics could help to safeguard and maintain the sensitivity profile for a longer user [30].

This study also established some specific clinical syndromes associated with highly resistant pathogens. For instance, recurrent genital discharges from STI clients yielded Neisseria gonorrhoeae resistant to gentamycin and doxycycline but sensitive to ceftriaxone and ertapenem. The growing cases of recurrent infections in the STI department due to emerging gentamycin resistances have also been reported in other studies conducted in Malawi and sub-Saharan region [10, 21]. There is a growing concern that emerging resistances in STI causing pathogens may be perpetuated by antibiotics overuse and the syndromic management approach which rarely makes use of laboratory investigations to guide treatment [8, 31, 32]. It is therefore important to utilize microbiological investigations to guide treatment for all suspected recurrent or re-infections in patients returning to the facility, few days or weeks after the initial STI treatment.

Among the staphylococcus sp detected were the methicillin resistant staphylococcus aureus (MRSA) defined as an oxacillin minimum inhibitory concentration (MIC) of greater than or equal to 4 micrograms/Ml [33]. The MRSA expressed resistance towards cephalosporins, floroquinolones, penicillins and macrolides but susceptible to vancomycin, chloramphenicol, amikacin, and nitrofurantoin. The findings are supported by other previous studies which also found MRSA expressing 100% resistance to penicillins such as ampicillin and 100% sensitivity to vancomycin [16]. MRSA infection is one of the leading causes of hospital-acquired infections associated with significant morbidity, mortality, and cost burden [33].

It is quite concerning to note that strong antibiotics such as Tigecycline that have shown 100% sensitivity over all resistant isolates are not included in the Malawi essential medicines list. This is a set back as clinicians will continue to prescribe less effective antibiotics based on availability or affordability. In that regard, hospital management team as well as drug and therapeutic committees (DTC)  have a role to play in ensuring that effective antibiotics recommended by AMS teams are available to avoid unnecessary alternative treatments which may predispose patients to sub-optimal concentrations, a driver of future MDR infections [14, 22].

The findings of this study highlights a high burden of AMR driven my multiple factors. Among them are high empiric use of antibiotics due to limited diagnostic capacity and high selection of watch antibiotics for conditions that can be treated by access antibiotics which have lower risk of inducing antibiotic resistance. This is against the WHO recommendation which states that at least 60% of prescribed antibiotics should be from the access group [34]. Other challenges of antibiotic use in Malawi include failure to update treatment guidelines so that it is in line with the prevailing resistances. For instance, at the time of this study, the latest treatment guideline in Malawi was published in 2015.

### Study limitation

We acknowledge that the absence of essential antibiotics during PPS might have affected the outcome interms of the reported frequencies in antibiotics use. It was also difficult to determine whether all antibiotics tested over the past four years maintained their effectiveness or not as they were subjected to varying storage conditions. Besides, both the patient and AST test results sample sizes were comparatively small to other referred studies done in hospital settings. This is because we only targeted suspected MDR cases on return patients and those with prolonged hospital stay. As such the PPS findings on the pattern of antimicrobial use may not be a true reflection of the hospital prescribing practices which are usually dynamic. Moreover, few facilities participated at national level hence findings did not reflect national level. However, the combination of the PPS findings, the patents antibiotic history as well as laboratory AMR data improved the validity and reliability of the study outcomes.

## Conclusion

In this study, we recognized a problem of multidrug resistance in patients experiencing clinical failure in a variety of clinical syndromes such as genital discharges, dysuria, abscess and persistent temperatures. The findings underline the need for strengthening antimicrobial stewardship programmes such AMR surveillance. With the current first line antibiotics being rendered ineffective, and the overuse of essential antibiotics including ceftriaxone, there is need for a quick review of the available standard treatment guidelines, taking into consideration the emerging multi-drug resistances. Hospital stewardship programmes should therefore take a leading role in safeguarding antibiotics through establishment of measurable antimicrobial use targets, aiming at reducing the use of broad-spectrum antibiotics in the watch list, complying with the country specific treatment guidelines and revamping microbiology labs to increase the uptake of antimicrobial susceptibility testing to guide antibiotic prescriptions.

### Recommendation

Considering changing behavior is crucial, we propose further qualitative and behavioral studies in-order to further explore barriers and facilitators relating to healthcare workers capability, opportunity and motivation to change. This will ensure that the AMS behavior change interventions for improving prescribing practices are not only robust but also sustainable. We further recommend control of use of watch and reserve antibiotics through policy and adopting up to date clinical and practices.

## Data Availability

The data used in this study are available from the corresponding author (Brany Mithi, Email: branytitus@gmail.com) upon reasonable request.

## Footnotes

1 Overall hospital antimicrobial prevalence in adult wards was at 75.0% which was quite high compared with 32% at national level. The prevalence was much higher in children ward compared compare to adults (83.3% vs. 75.0%). Common diagnoses treated with therapeutic antibiotics include sepsis and lower respiratory tract infection (pneumonia).

## Abbreviation

AMR: Antimicrobial Resistances
AST: Antimicrobial Susceptibility Testing
COM-B: Capability Opportunity Motivation Behavior
EUCAST: European Committee on Antimicrobial Susceptibility Testing
GLASS: Global Antimicrobial Resistance and Use Surveillance System
GPPS: Global Point Prevalent Survey
HCWs: Health Care Workers
HIV: Human Immunodeficiency Virus
LMIC: Low and Middle Income Countries
MDR: Multi-Drug Resistance
MoH: Ministry of Health
NHSRC: National Health Science Research Committee
PHIM: Public Health Institute of Malawi
SADCAS: Southern Africa Development Community Accreditation Services
WHO: World Health Organization
